# β-Lactamase diversity in *Acinetobacter baumannii*

**DOI:** 10.1128/aac.00784-24

**Published:** 2025-02-10

**Authors:** Andrew R. Mack, Andrea M. Hujer, Maria F. Mojica, Magdalena A. Taracila, Michael Feldgarden, Daniel H. Haft, William Klimke, Arjun B. Prasad, Robert A. Bonomo

**Affiliations:** 1 Department of Molecular Biology and Microbiology, Case Western Reserve University School of Medicine 12304 , Cleveland, Ohio, USA; 2 Research Service, Louis Stokes Cleveland Department of Veterans Affairs Medical Center, Cleveland, Ohio, USA; 3 Department of Medicine, Case Western Reserve University School of Medicine 12304 , Cleveland, Ohio, USA; 4 CWRU-Cleveland VAMC Center for Antimicrobial Resistance and Epidemiology (Case VA CARES) 2546 , Cleveland, Ohio, USA; 5 National Center for Biotechnology Information, National Library of Medicine, National Institutes of Health 2511 , Bethesda, Maryland, USA; 6 Department of Pharmacology, Case Western Reserve University School of Medicine 12304 , Cleveland, Ohio, USA; 7 Department of Biochemistry, Case Western Reserve University School of Medicine 12304 , Cleveland, Ohio, USA; 8 Department of Proteomics and Bioinformatics, Case Western Reserve University School of Medicine 12304 , Cleveland, Ohio, USA; 9 Clinician Scientist Investigator, Louis Stokes Cleveland Department of Veterans Affairs Medical Center, Cleveland, Ohio, USA; University of Fribourg, Fribourg, Switzerland

**Keywords:** antibiotic resistance, *Acinetobacter baumannii*, beta-lactamases, bioinformatics

## Abstract

*Acinetobacter baumannii* is a clinically important, Gram-negative pathogen responsible for a wide variety of nosocomial and community-acquired infections. Antibiotic resistance is a serious concern, as the organism has a wide variety of intrinsic resistance mechanisms, including chromosomal class C (*bla*_ADC_) and D (*bla*_OXA-51_ family) β-lactamases, and the ability to readily acquire additional β-lactamases. Surveillance studies can reveal the diversity and distribution of β-lactamase alleles, but are difficult and expensive to conduct. Herein, we describe an approach using publicly available data derived from whole genome sequences, to explore the diversity and distribution of β-lactamase alleles across 28,330 isolates. The most common intrinsic alleles at the time of writing were *bla*_ADC-73_, *bla*_ADC-30_, *bla*_ADC-222_, *bla*_ADC-33_, and *bla*_OXA-66_, and the most common acquired allele was *bla*_OXA-23_. Interestingly, only 63.0% of assigned *bla*_ADC_ alleles were encountered and the 10 most common *bla*_ADC_ and intrinsic *bla*_OXA_ alleles represented approximately 75% of their respective gene totals while dozens were extremely infrequent. Differences were observed over time and geography. Surprisingly, more distinct unassigned (i.e., lacking a *bla*_ADC_ or *bla*_OXA_ number) alleles were encountered than distinct, assigned alleles. Understanding the diversity and distribution of β-lactamase alleles helps to prioritize variants for further research, selects targets for drug development, and may aid in selecting therapies for a given infection.

## INTRODUCTION

*Acinetobacter baumannii* is a clinically important, Gram-negative pathogen responsible for a wide variety of both nosocomial and community-acquired infections, including respiratory tract, bloodstream, bone, urinary tract, wound, and meningeal infections (1, 2), and commonly harbors high levels of antibiotic resistance (3). Carbapenem-resistant *A. baumannii* (CRAb) is considered a “critical priority pathogen” by the World Health Organization (WHO) (4) and an “urgent threat” by the Centers for Disease Control and Prevention (CDC). CRAb was responsible for 700 deaths and $281 million in healthcare costs in the United Sates in 2017 (5). Likely driven by the coronavirus disease 2019 pandemic, rates of CRAb increased 35% overall from 2019 to 2020 (including a 78% increase in hospital-onset cases) (6).

Unfortunately, treatment is often complicated by intrinsic resistance mechanisms. *A. baumannii* can be notoriously difficult for drugs to penetrate, as outer membrane porin components possess 70-fold lower pore-forming activity than *Escherichia coli* and *Pseudomonas aeruginosa* equivalents, and outer membrane permeability to some antibiotics is 100-fold lower than for *E. coli* (7). Commonly isolated in the nosocomial environment, but also found in both environmental and animal reservoirs, *A. baumannii* (along with other *Acinetobacter* species) possesses a high capacity to acquire additional resistance genes (8) and have been described as “potential reservoirs and dispensers of the resistance determinants, particularly in the hospital environments” (9).

While many recent analyses have examined the prevalence of β-lactamases in *A. baumannii* (10–14), these studies tend to include a relatively limited sample size (in terms of both number and distribution of isolates). In addition, many have a specific focus (e.g., carbapenemase genes), are published several years after data collection was completed, and do not typically make extensive use of whole genome sequencing (WGS) to explore the resistome. A recently created *A. baumannii* (15) diversity panel captures a wide variety of β-lactamase alleles and other resistance determinants, and represents an important strain collection for research and development, but is not designed to provide dynamic snapshots of the complex allelic diversity.

Given the relative lack of broad surveys of β-lactamase alleles in *A. baumannii* and inspired by the success of our recent effort analyzing the genomes of 98 CRAb isolates collected as part of the Primers III study to provide a contemporary snapshot of β-lactamases in *A. baumannii* (16), we set out to gain a better understanding of the genetic landscape of this complex and diverse species by examining the β-lactamase alleles of 28,330 isolates found in the National Center for Biotechnology Information (NCBI) Pathogen Detection databases (https://www.ncbi.nlm.nih.gov/pathogens/). This data set allows for the examination of a wide array of variables, including geographic and temporal, over which to track changes in the β-lactamase resistome and is updated in near real time (17), potentially allowing changes to be quickly observed moving forward. Understanding the genetic diversity of β-lactamases in *A. baumannii* will help drive innovation by (i) “prioritizing” β-lactamase variants for further biochemical and microbiological studies to understand changing/evolving resistance mechanisms; (ii) helping select targets for the design, optimization, and testing of new β-lactam antibiotics and β-lactamase inhibitors; (iii) potentially helping guide antibiotic selection for infections with a well-defined β-lactamase resistome; and (iv) assessing the impact of antibiotic and natural selection on bacterial populations and how this shapes their evolution.

## RESULTS

### Overview of data set

As of 9 September 2024 MicroBIGG-E includes 30,586 distinct *Acinetobacter* isolates covering seven *Acinetobacter* species (all of which are among the 72 species that can be differentiated by the Pasteur MLST scheme [18]) and 58 isolates for which a published species has not been assigned. Filtering to include only full-length, high-quality *bla* (β-lactamase) genes in *Acinetobacter baumannii*, deduplicated at the isolate level, leads to a final data set of 28,330 isolates encoding 93,166 *bla* genes (Table S1).

As anticipated, *bla*_ADC_ (28,940 genes) and intrinsic *bla*_OXA_ (*bla*_OXA-51_ family members; 28,461 genes) were the most frequently encountered *bla* gene families, representing 61.6% of total *bla* genes, with 11 acquired *bla*_OXA_ families and 14 additional acquired gene families occurring in five or more isolates. For the purposes of this analysis, “assigned” refers to a distinct *bla* allele that has been designated with an identifying number (e.g., *bla*_ADC-7_); “unassigned” *bla* alleles do not have a specific number attributed to that *bla* gene. A total of 568 distinct, assigned *bla* alleles were present, including 203 distinct, assigned *bla*_ADC_ alleles and 269 distinct, assigned, intrinsic *bla*_OXA_ alleles. Collectively, 1,133 distinct unassigned alleles were present, including 728 distinct, unassigned *bla*_ADC_ alleles (234 appearing in two or more isolates) and 252 distinct, unassigned, intrinsic *bla*_OXA_ alleles (51 appearing in two or more isolates) (Table S2).

### Origin of isolates

#### Geographic

**Fig 1:**
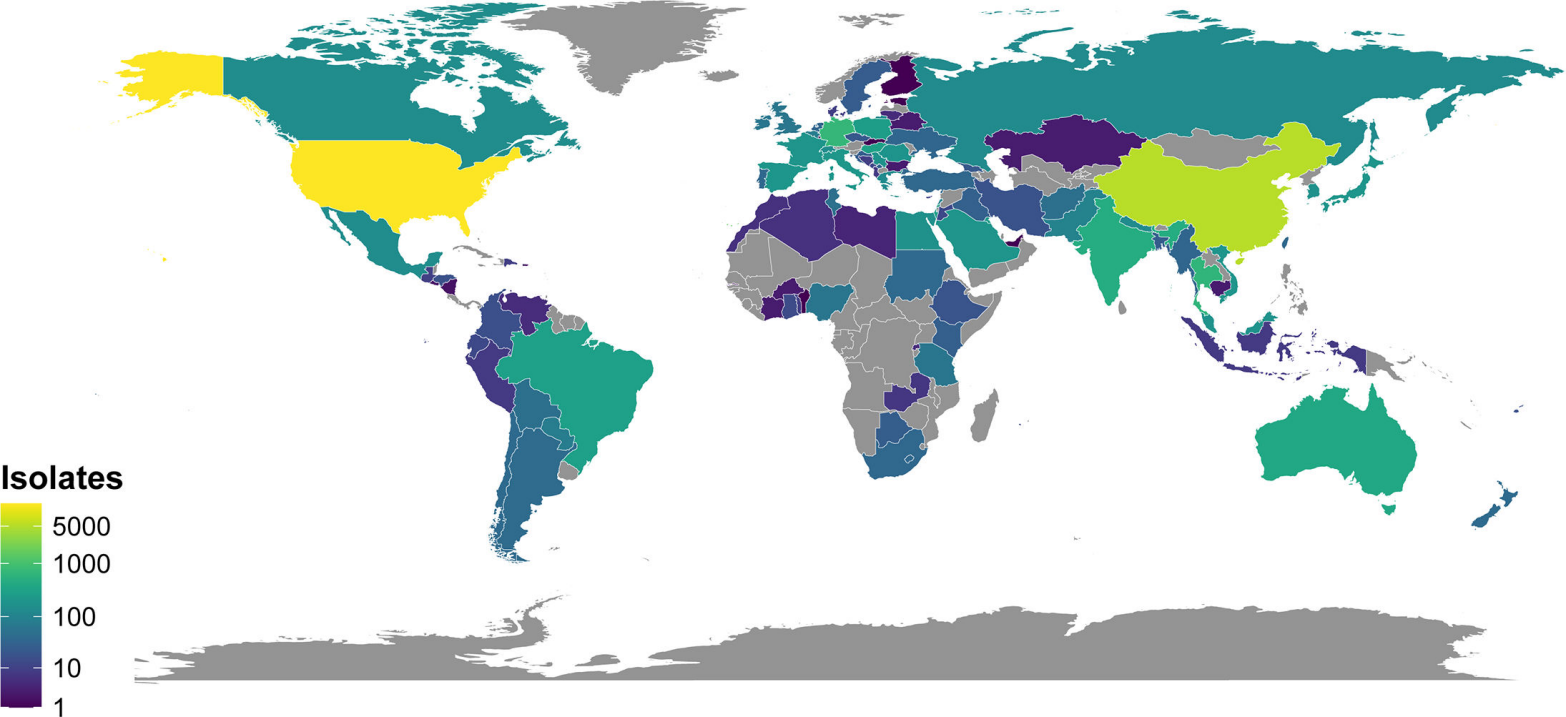
Geographic origin of isolates used in the analysis. Geographic origin was unavailable for 1,485 of 28,330 isolates. Gray indicates that no isolates were analyzed from a given country/region. The map was created using ggplot2 package for R.

In total, 104 countries are represented, spanning all regions examined (Fig. 1). The USA and China contributed the most isolates (46.7% and 18.4%, respectively) while a total of 38 countries contributed 50 or more isolates each (Table S3). Regionally, North America, East & Southeast Asia, and Europe & Central Asia contributed the most isolates (Table S3; Fig. S1). Generally, higher- and middle-income countries are better represented, and lower-income countries are less well represented.

#### Collection date

Both historic and contemporary isolates are included, with the vast majority collected in recent years as large-scale collection and sequencing has become more frequent. Overall, 95 isolates were collected prior to 2000 and a mean of 2,210 isolates collected annually from 2015 to 2023 (Table S4).

### β-Lactamase (*bla*) genes in *A. baumannii*

Among the 26,109 assigned *bla*_ADC_ genes present, we identified 203 distinct alleles. Of these, only 11 appeared in 1.0% or more of isolates while an additional 8 appeared in 0.5% to 1.0% of isolates. The four most frequent alleles, occurring in 3.0% or more of isolates, were *bla*_ADC-73_ (7,962 isolates, 28.1%), *bla*_ADC-30_ (7,126 isolates, 25.2%), *bla*_ADC-222_ (1,611 isolates, 5.7%), and *bla*_ADC-33_ (979 isolates, 3.5%) (Table 1). In contrast, 103 distinct, assigned alleles appear in 10 or fewer isolates and 43 appear in only a single isolate (Table S5).

**TABLE 1:** *A. baumannii bla*_ADC_ (left) and intrinsic *bla*_OXA_ (right) alleles occurring in 100 or more isolates^*a*^

Allele	Isolates	Proportion	Rank	Allele	Isolates	Proportion	Rank
*bla*_ADC-73_	7,962	28.1%	1	*bla*_OXA-66_	17,328	61.2%	1
*bla*_ADC-30_	7,126	25.2%	2	*bla*_OXA-95_	1,598	5.6%	2
*bla*_ADC-222_	1,611	5.7%	3	*bla*_OXA-82_	1,389	4.9%	3
*bla*_ADC-33_	979	3.5%	4	*bla*_OXA-69_	1,188	4.2%	4
*bla*_ADC-26_	610	2.2%	5	*bla*_OXA-64_	606	2.1%	5
*bla*_ADC-79_	527	1.9%	6	*bla*_OXA-65_	511	1.8%	6
*bla*_ADC-25_	482	1.7%	7	*bla*_OXA-91_	408	1.4%	7
*bla*_ADC-52_	392	1.4%	8	*bla*_OXA-71_	345	1.2%	8
*bla*_ADC-56_	345	1.2%	9	*bla*_OXA-51_	288	1.0%	9
*bla*_ADC-11_	319	1.1%	10	*bla*_OXA-223_	238	0.8%	10
*bla*_ADC-76_	296	1.0%	11	*bla*_OXA-68_	238	0.8%	10
*bla*_ADC-268_	270	1.0%	12	*bla*_OXA-100_	185	0.7%	11
*bla*_ADC-223_	257	0.9%	13	*bla*_OXA-120_	170	0.6%	12
*bla*_ADC-162_	256	0.9%	14	*bla*_OXA-402_	163	0.6%	13
*bla*_ADC-74_	210	0.7%	15	*bla*_OXA-98_	162	0.6%	14
*bla*_ADC-199_	182	0.6%	16	*bla*_OXA-90_	156	0.6%	15
*bla*_ADC-169_	174	0.6%	17	*bla*_OXA-104_	148	0.5%	16
*bla*_ADC-191_	147	0.5%	18	*bla*_OXA-829_	136	0.5%	17
*bla*_ADC-82_	142	0.5%	19	*bla*_OXA-834_	129	0.5%	18
*bla*_ADC-155_	141	0.5%	20	*bla*_OXA-259_	126	0.4%	19
*bla*_ADC-156_	135	0.5%	21	*bla*_OXA-113_	114	0.4%	20
*bla*_ADC-152_	133	0.5%	22				
*bla*_ADC-176_	126	0.4%	23				
*bla*_ADC-182_	124	0.4%	24				
*bla*_ADC-212_	124	0.4%	24				
*bla*_ADC-263_	119	0.4%	25				
*bla*_ADC-87_	118	0.4%	26				
*bla*_ADC-158_	114	0.4%	27				
*bla*_ADC-32_	108	0.4%	28				

^*a*^ Percentage represents the percentage of isolates encoding a specific allele.

Unassigned alleles appear more diverse than assigned alleles, with 728 distinct *bla*_ADC_ alleles occurring a total of 2,329 times in the data set. Four unassigned alleles occur in 0.2% or more of isolates: RefSeq (19) protein accession number WP_125147065.1 (156 isolates, 0.55%), WP_163063065.1 (87 isolates, 0.31%), WP_001211234.1 (65 isolates, 0.22%), and WP_001211230.1 (62 isolates, 0.22%) (Table S6).

Among the 27,696 assigned, intrinsic *bla*_OXA_ genes present, we identified 252 distinct alleles. The two most frequent were *bla*_OXA-66_ (17,328 isolates, 61.2%) and *bla*_OXA-95_ (1,598 isolates, 5.6%) with all other alleles occurring in less than 5% of isolates. A total of nine intrinsic *bla*_OXA_ alleles appeared in 1.0% or more of isolates (Table 1) and eight in 0.5% to 1.0% of isolates. In contrast, 203 distinct alleles occurred in 10 or fewer isolates and 95 in only a single isolate (Table S5).

A total of 680 unassigned, intrinsic *bla*_OXA_ genes correspond to 252 distinct alleles. Two unassigned alleles occur in 0.2% or more of isolates: WP_202746419.1 (90 isolates or 0.32%) and EHU1184883.1 (60 isolates, 0.21%). In contrast, 331 alleles occurred in 10 or fewer isolates and 206 in only a single isolate (Table S6).

Overall, 36,080 acquired β-lactamase genes are present, with 96 distinct alleles corresponding to 11 acquired *bla*_OXA_ families and 14 additional gene families. The most frequent acquired gene is the *bla*_OXA-23_ family (18,102 isolates) and the second most is *bla*_TEM_ (8,864 isolates) with eight others occurring 100 or more times each: the *bla*_OXA-24_ family, *bla*_NDM_, *bla*_CARB_, the *bla*_OXA-134_ family, *bla*_PER_, the *bla*_OXA-58_ family, *bla*_CTX-M_, and *bla*_GES_ (Table S7). Aside from *bla*_NDM_, of which *bla*_NDM-1_ (1,483 alleles, 5.2%), *bla*_NDM-2_ (39 isolates, 0.14%), and *bla*_NDM-5_ (27 isolates, 0.10%) were the most common, metallo-β-lactamases are quite infrequent in the data set with *bla*_VIM_ (13 isolates, 0.05%) and *bla*_IMP_ (six isolates, 0.02%) being the only other examples encountered (Table S2). As with intrinsic alleles, substantial numbers of unassigned alleles are present for many acquired alleles, particularly the *bla*_OXA-23_ family (12 distinct, assigned and 54 distinct unassigned alleles), *bla*_TEM_ (8 distinct, assigned and 40 distinct unassigned alleles), and the *bla*_OXA-24_ family (7 distinct, assigned alleles and 22 distinct unassigned alleles) (Table S2).

### Allele frequency by region

**Fig 2:**
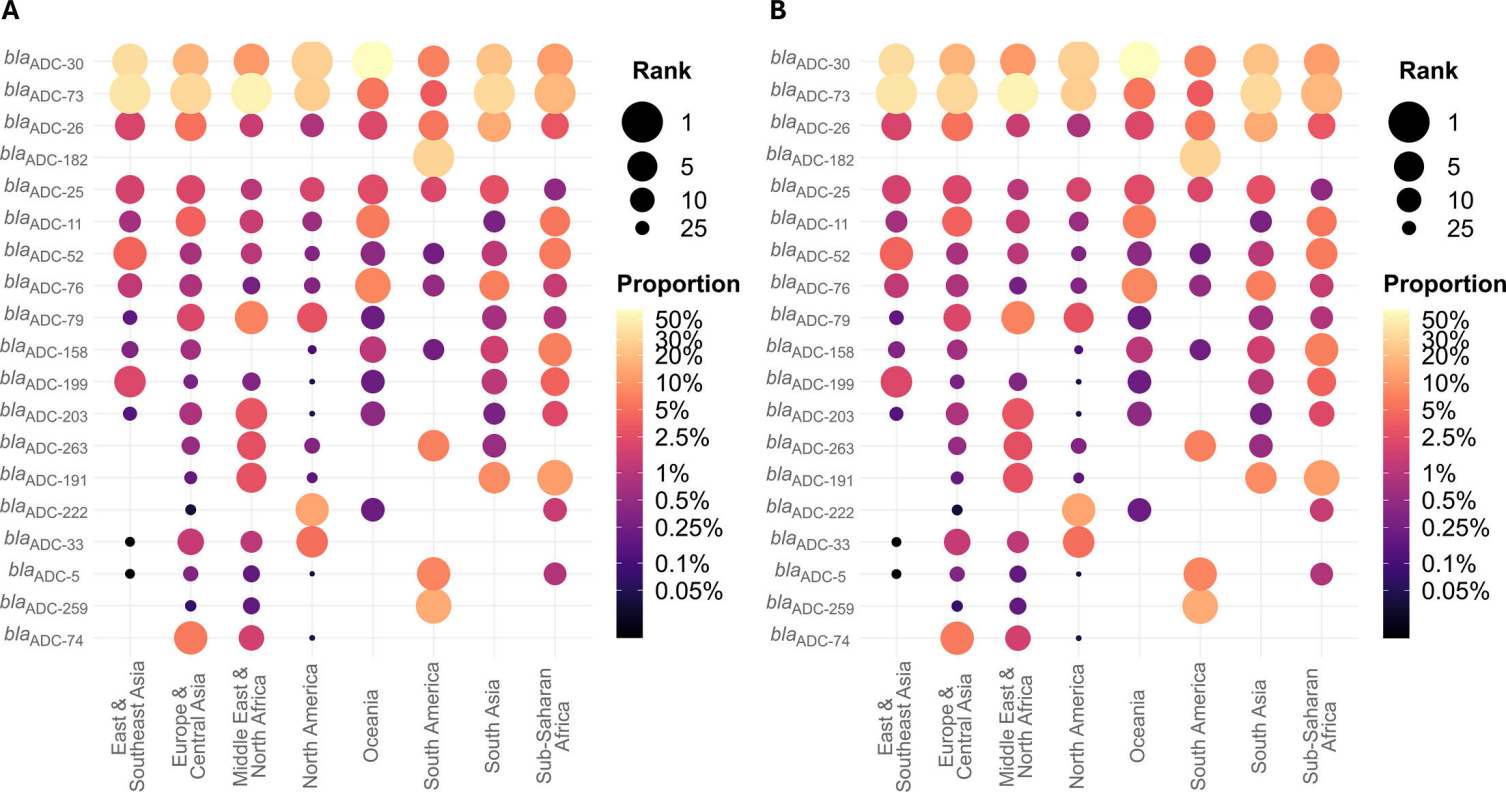
*A. baumannii* alleles by region. Rank and proportion of the five most common (**A**) *bla*_ADC_ (left) and (**B**) intrinsic *bla*_OXA_ alleles in *A. baumannii* isolates from each region across all regions. Color indicates proportion of isolates containing a given allele and size indicates the frequency rank of an allele in each region.

Among *bla*_ADC_ alleles, the most frequent is *bla*_ADC-73_ in all regions except North America (second most frequent), Oceania (fourth most frequent), and South America (eighth most frequent). The most frequent in North America and Oceania are *bla*_ADC-30_, which is the second most frequent in all other regions except South America (fourth most frequent) (Fig. 2A; Table S8). The most frequent allele in South America is *bla*_ADC-182_, which is found in 124 isolates (30.2%) but does not appear in any other region. Additionally, *bla*_ADC-259_ is the second most frequent allele in South America (61 isolates, 14.8%), but is otherwise only found in two isolates each from Europe & Central Asia and Middle East & North Africa.

Among intrinsic *bla*_OXA_ alleles, the most frequent is *bla*_OXA-66_ in all regions except South America (fifth most frequent; *bla*_OXA-65_ is most frequent) and Sub-Saharan Africa (second most frequent; *bla*_OXA-69_ is most frequent) (Fig. 2B; Table S9). Among acquired *bla*_OXA_ alleles, *bla*_OXA-23_ (*bla*_OXA-23_ family) is by far the most common across all regions.

Despite being the most common acquired allele across all regions, regional prevalence of *bla*_OXA-23_ and family members varies widely, ranging from as low as 47.0% of isolates in Europe & Central Asia region to as high as 85.7% of isolates in South Asia (Table S10).

### Allele frequency over time

**Fig 3:**
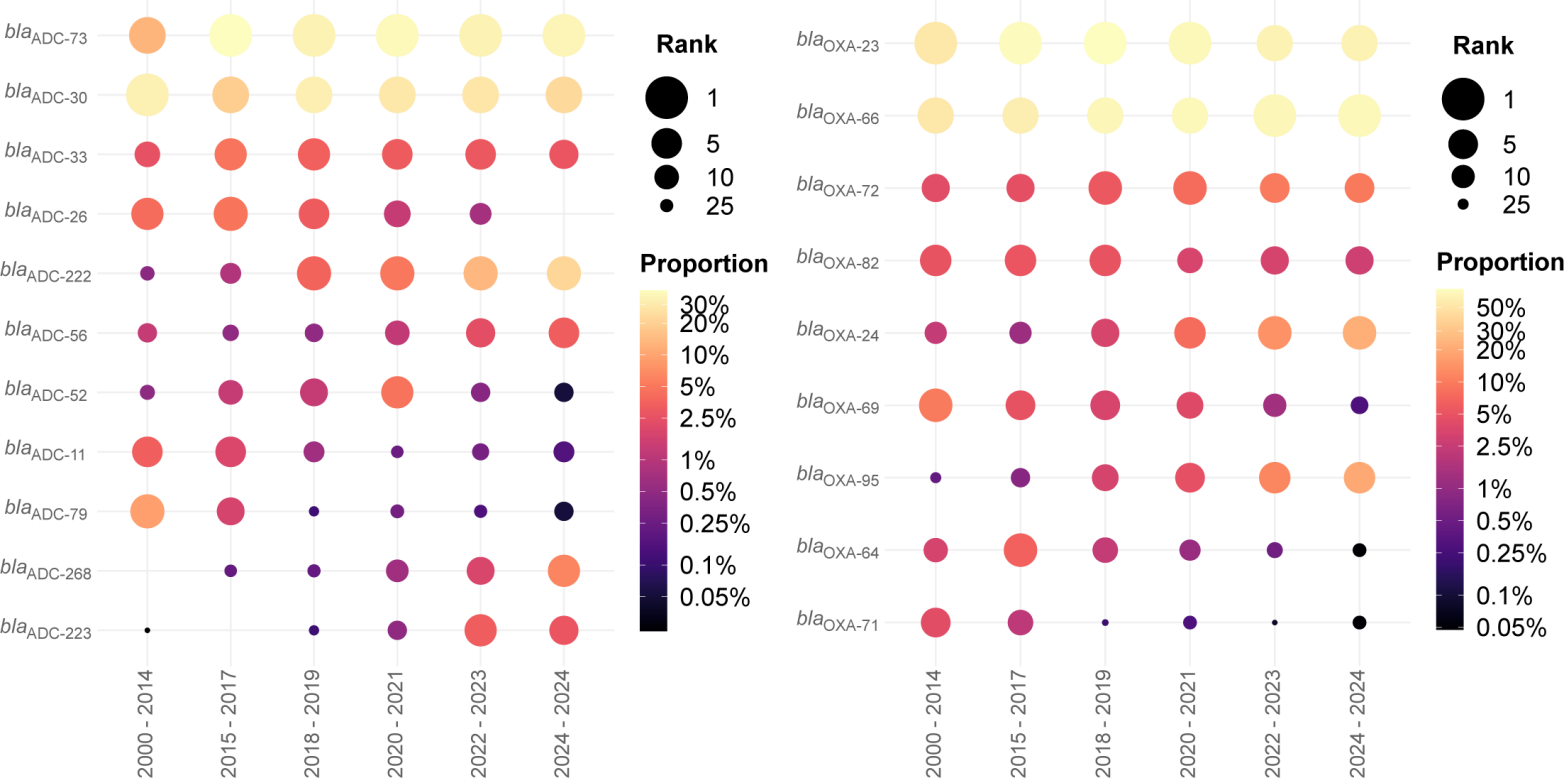
*A. baumannii* alleles by collection period. Rank and proportion of the five most common (**A**) *bla*_ADC_ and (**B**) intrinsic *bla*_OXA_ alleles in *A. baumannii* isolates from each collection across all periods. Color indicates proportion of isolates containing a given allele and size indicates the frequency rank of an allele in each period.

The two most common *bla*_ADC_ alleles overall are reasonably consistent with time (*bla*_ADC-30_ was most common in 2000–2014 and second most common since, while *bla*_ADC-73_ was second most common in 2000–2014 and has been most common in all subsequent periods). Many alleles have emerged over time, including *bla*_ADC-56_ (16th most common in 2000–2014 increasing to sixth in 2022–2023 and fifth in 2024), *bla*_ADC-222_ (23rd and 14th most common in 2000–2014 and 2015–2017, respectively, but third most common since), and *bla*_ADC-223_ (increasing from a single isolate in 2000–2014 to fourth most common in 2022–2023 and sixth in 2024). Conversely, *bla*_ADC-79_ decreased from third most common in 2000–2014 and seventh in 2015–2017, and no higher than 16th since (Fig. 3A; Table S11).

Among intrinsic *bla*_OXA_ alleles, *bla*_OXA-66_ has remained the most common across all periods since 2000. In contrast, *bla*_OXA-69_ decreased from second most common in 2000–2014 to ninth most common in 2024, *bla*_OXA-71_ decreased from fourth most common in 2000–2014 and sixth most common in 2015–2017 to no more than 14th most common in any subsequent period, *bla*_OXA-95_ increased from 18th most common in 2000–2014 to second most common in all periods since 2020, and *bla*_OXA-223_ increased from 26th most common in 2000–2014 to fourth most common in 2022–2023 and fifth in 2024 (Fig. 3B; Table S12). Interestingly, the most common acquired *bla*_OXA_ alleles appear more consistent while less common alleles seem to more quickly arise and disappear: *bla*_OXA-23_ (*bla*_OXA-23_ family) the most common and *bla*_OXA-72_ (*bla*_OXA-24_ family) the second or third most common across all periods since 2000; *bla*_OXA-253_ (*bla*_OXA-143_ family) decreased from sixth most common in 2000–2014 to 10th or 11th between 2015 and 2021, but has not been observed since; *bla*_OXA-164_ (*bla*_OXA-58_ family) was 10th most common in 2000–2014 but has not been observed since; and *bla*_OXA-252_ (*bla*_OXA-48_ family) and *bla*_OXA-139_ (*bla*_OXA-24_ family) were not observed through 2019 but have been between seventh and ninth most common in all periods since 2020.

### Allele combinations

Disproportionate allele frequencies carry into allele combinations, with just the 11 distinct combinations occurring in 1.0% or more of isolates representing a combined 52.1% of isolates. The most frequent combinations are *bla*_ADC-73_/*bla*_OXA-23_/*bla*_OXA-66_ /*bla*_TEM-1_ (4,380 isolates, 15.5%), *bla*_ADC-73_/*bla*_OXA-23_/*bla*_OXA-66_ (2,875 isolates, 10.1%), *bla*_ADC-30_/*bla*_OXA-23_/*bla*_OXA-66_/*bla*_TEM-1_ (1,869 isolates, 6.6%), and *bla*_ADC-30_/*bla*_OXA-23_/*bla*_OXA-66_ (1,679 isolates, 5.9%) (Table 2). In contrast, 1,664 combinations occur in 10 or fewer isolates and including 915 in only a single isolate (Table S13). Among the above alleles, *bla*_OXA-23_ is a member of the *bla*_OXA-23_ family and *bla*_OXA-66_ is a member of the *bla*_OXA-51_ family.

**TABLE 2:** Allele combinations occurring in 100 or more *A. baumannii* isolates^*a*^

Combination of alleles	Isolates	Proportion
*bla*_ADC-73_, *bla*_OXA-23_, *bla*_OXA-66_, *bla*_TEM-1_	4,380	15.5%
*bla*_ADC-73_, *bla*_OXA-23_, *bla*_OXA-66_	2,875	10.1%
*bla*_ADC-30_, *bla*_OXA-23_, *bla*_OXA-66_, *bla*_TEM-1_	1,869	6.6%
*bla*_ADC-30_, *bla*_OXA-23_, *bla*_OXA-66_	1,679	5.9%
*bla*_ADC-30_, *bla*_OXA-66_, *bla*_OXA-72_	872	3.1%
*bla*_ADC-222_, *bla*_OXA-24_, *bla*_OXA-95_	813	2.9%
*bla*_ADC-33_, *bla*_OXA-23_, *bla*_OXA-82_	613	2.2%
*bla*_ADC-30_, *bla*_NDM-1_, *bla*_OXA-23_, *bla*_OXA-66_	458	1.6%
*bla*_ADC-222_, *bla*_OXA-23_, *bla*_OXA-95_	436	1.5%
*bla*_ADC-30_, *bla*_OXA-24_, *bla*_OXA-66_	408	1.4%
*bla*_ADC-30_, *bla*_OXA-66_, *bla*_TEM-1_	344	1.2%
*bla*_ADC-33_, *bla*_OXA-82_	264	0.9%
*bla*_ADC_, *bla*_OXA-23_, *bla*_OXA-66_	227	0.8%
*bla*_ADC-223_, *bla*_OXA-23_, *bla*_OXA-66_	222	0.8%
*bla*_ADC-222_, *bla*_CARB-2_, *bla*_OXA-23_, *bla*_OXA-95_	203	0.7%
*bla*_ADC-73_, *bla*_NDM-1_, *bla*_OXA-23_, *bla*_OXA-66_	185	0.7%
*bla*_ADC_, *bla*_OXA-23_, *bla*_OXA-66_, *bla*_TEM-1_	179	0.6%
*bla*_ADC-162_, *bla*_OXA-23_, *bla*_OXA-66_, *bla*_TEM-1_	161	0.6%
*bla*_ADC-30_, *bla*_OXA-66_, *bla*_OXA-237_, *bla*_TEM-1_	155	0.5%
*bla*_ADC_, *bla*_OXA_	154	0.5%
*bla*_ADC-30_, *bla*_OXA-66_, *bla*_OXA-235_	149	0.5%
*bla*_ADC-79_, *bla*_OXA-71_	148	0.5%
*bla*_ADC-268_, *bla*_OXA-23_, *bla*_OXA-66_, *bla*_TEM-1_	147	0.5%
*bla*_ADC-30_, *bla*_OXA-66_	147	0.5%
*bla*_ADC-74_, *bla*_OXA-66_, *bla*_OXA-72_	141	0.5%
*bla*_ADC_, *bla*_OXA-24_, *bla*_OXA-66_	140	0.5%
*bla*_ADC-30_, *bla*_OXA-66_, *bla*_OXA-237_	129	0.5%
*bla*_ADC_, *bla*_OXA-24_, *bla*_OXA-829_	129	0.5%
*bla*_ADC-155_, *bla*_OXA-69_	126	0.4%
*bla*_ADC-56_, *bla*_OXA-66_, *bla*_OXA-72_	112	0.4%
*bla*_ADC_, *bla*_OXA-66_, *bla*_OXA-72_	108	0.4%
*bla*_ADC-268_, *bla*_OXA-23_, *bla*_OXA-66_	106	0.4%
*bla*_ADC-52_, *bla*_ADC-199_, *bla*_CARB-16_, *bla*_NDM-1_, *bla*_OXA-23_, *bla*_OXA-91_	105	0.4%
*bla*_ADC-156_, *bla*_OXA-120_	102	0.4%

^*a*^ Percentage represents the percentage of isolates encoding a specific allele. The inclusion of “*bla*_ADC_” or “*bla*_OXA_” without a number collectively refers to all unassigned alleles of the corresponding gene, which are comprised of many unique variants.

Examining only intrinsic alleles, the most frequent combinations are *bla*_ADC-73_/*bla*_OXA-66_ (7,819 isolates, 27.6%) and *bla*_ADC-30_/*bla*_OXA-66_ (6,746 isolates, 23.8%). An additional four combinations occur in 1.0% to 5.0% of isolates while 694 occur in 10 or fewer isolates and 383 in only a single isolate (Table S14).

### Carbapenemases

Given the scope of this analysis, we did not examine the presence of the upstream IS*Aba*1 insertion elements associated with intrinsic *bla*_OXA-51_ family overexpression and carbapenemase activity (20) as this information is not directly included in the Pathogen Detection databases. We note, however, that the well-studied carbapenemase allele *bla*_OXA-82_ (characterized by the L167V substitution) (21) was the third most common intrinsic *bla*_OXA_ allele overall, found in 1,389 isolates. Among acquired carbapenemases, the most common was the *bla*_OXA-23_ family (18,102 isolates, 63.9%), followed by the *bla*_OXA-24_ family (3,983 isolates, 14.1%), *bla*_NDM_ (1555 isolates, 5.5%), the *bla*_OXA-134_ family (639 isolates, 2.3%), the *bla*_OXA-58_ family (418 isolates, 1.5%), the *bla*_OXA-143_ family (43 isolates, 0.15%), the *bla*_OXA-48_ family (31 isolates, 0.11%), *bla*_KPC_ (13 isolates, 0.05%), *bla*_VIM_ (13 isolates, 0.05%), *bla*_IMP_ (6 isolates, 0.02%), and *bla*_GES_ (5 isolates, 0.02%) (Table S15).

**Fig 4:**
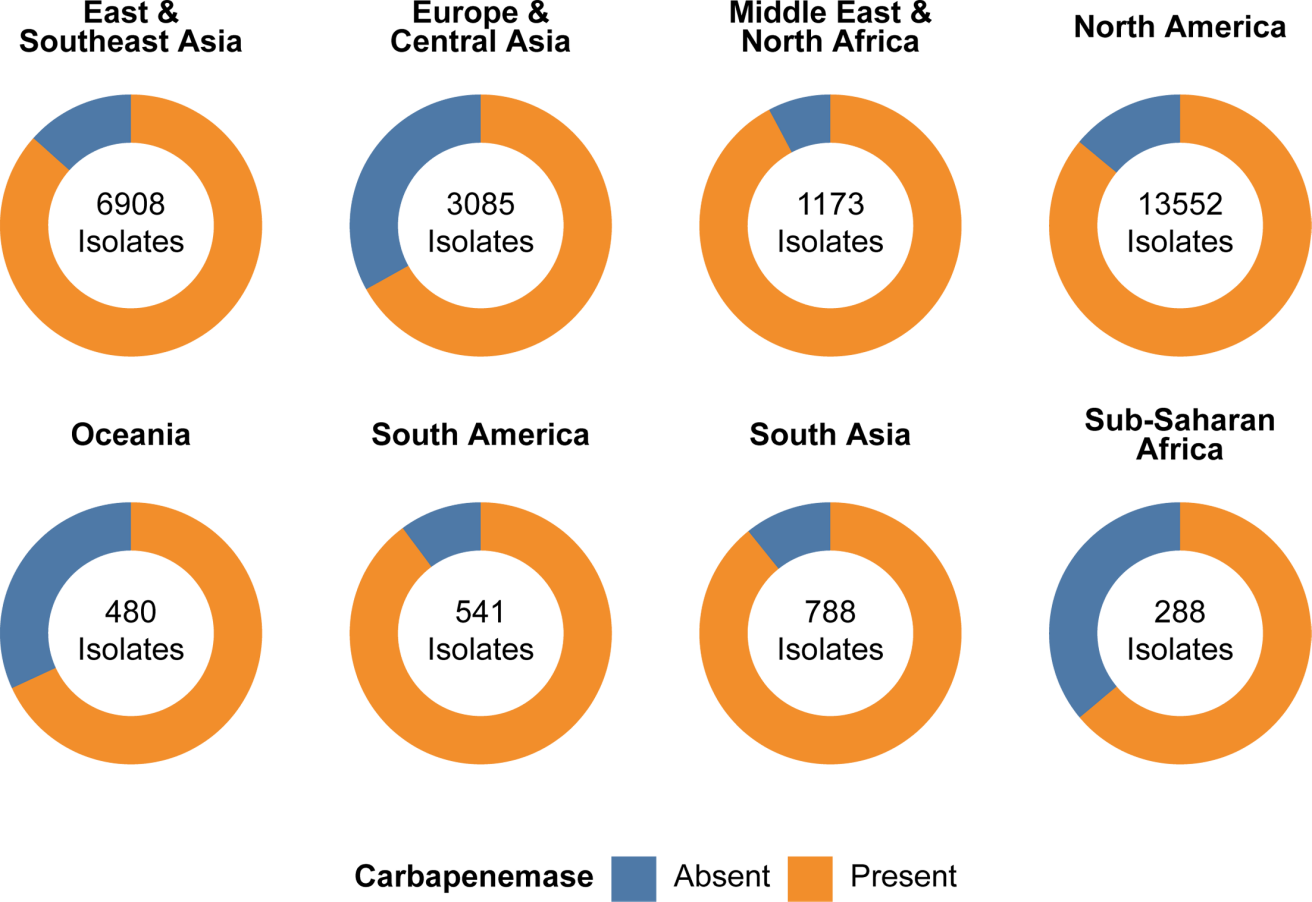
Proportion of isolates encoding carbapenemase genes by region. A total of *A. baumannii* isolates (81.2%) encode carbapenemase genes. For purposes of this analysis, *bla*_OXA-51_ family members were excluded.

Regionally, the proportion of isolates encoding acquired carbapenemase genes ranges from 66.9% in Europe & Central Asia to 92.2% in Middle East & North Africa (Table S16; Fig. 4). Concerningly, the global presence of carbapenemase genes appears to have increased over time, from 66.0% of isolates collected in 2000–2014 to 96.2% of isolates collected in 2024 (Table S17), although we would not be surprised if—and cannot rule out the possibility that—some portion of this increase comes from a bias to sequence carbapenemase-positive isolates as researchers have become increasingly focused on CRAb over time.

### Breadth of allelic diversity

#### Multilocus sequence typing (MLST) and pathogen detection SNP (PDS) cluster frequency

MLST (22) and whole genome multilocus sequence typing (wgMLST) (23) are two common approaches to determining relatedness of bacterial isolates. The former utilizes a carefully curated set of typically seven, slowly evolving, housekeeping genes to determine allele variants and generate sequence types (STs), while the latter utilizes WGS to examine a much larger set of genes to determine the relatedness of isolates (24). The NCBI Pathogen Detection pipeline utilizes wgMLST data to generate nearest-neighbor clusters with a 25-allele cutoff (https://www.ncbi.nlm.nih.gov/pathogens/pathogens_help/#data-processing-clustering). These clusters are assigned accessions starting with PDS, standing for Pathogen Detection SNP (single nucleotide polymorphism), which we refer to as PDS clusters. Note that PDS clusters can change over time and are not archived, so the exact accessions mentioned below pertain only to the specific snapshot of data used in this analysis (Table S1), but general patterns and trends should hold true across releases.

Using the Pasteur *A. baumannii* MLST scheme, the data set contains 858 distinct STs and 191 complete isolates not matching a known ST. The most frequent sequence types are ST2 (18,168 isolates, 64.1%) and ST499 (1,499 isolates, 5.3%), while just four other STs are present in 1.0% or more of isolates: ST1, ST25, ST164, and ST3 (Table S18). Similarly, the high prevalence of ST2 (correlating with international clone 2) has been observed in previous studies (25, 26). By PDS, the data set contains 1,417 distinct PDS clusters with 3,887 unclustered isolates assumed to be not closely related to any other isolate. The most frequent cluster is PDS000188899.22 (3,033 isolates, 10.7%) while six others occur in 2.0% or more of isolates (Table S19).

Changes in the frequency of STs were observed over time and geography. Among the three most frequent overall: ST2 is the most common across all periods; ST499 increased from 17th most common in 2000–2014 to second for all periods since 2018; and ST1 decreased from second to fourth most common through 2021 to 11th most common in 2022–2023 and 12th in 2024 (Table S20). Geographically, ST2 is the most common in all regions except Sub-Saharan Africa (second most common) and South America (fifth most common); ST499 was the second most common in North America, no higher than ninth most common in any other region, and not found at all in four regions; ST79 is the most common in South America but no higher than 10th in any other region (Table S21).

An overview of allele frequency when the data is “deduplicated” and examined for the presence of individual alleles by ST and PDS cluster, to better account for closely related isolates, is provided in the Supplemental Text and Tables S22 and S23.

#### MLST and PDS associations with intrinsic alleles

In the process of deduplication by Pasteur MLST and PDS, the question of whether sequence types and clusters are associated with specific alleles emerges. Among the more common STs in the data set: ST2 is associated with 50 distinct *bla*_ADC_ alleles and 24 distinct, intrinsic *bla*_OXA_ alleles; ST499 is associated with five distinct *bla*_ADC_ alleles and four distinct *bla*_OXA_ alleles; ST1 is associated with 27 distinct *bla*_ADC_ alleles and 10 distinct *bla*_OXA_ alleles; and ST406 is associated with 10 distinct *bla*_ADC_ alleles and six distinct *bla*_OXA_ alleles. Interestingly, some sequence types are far more diverse for one gene than the other: ST79 is associated with 11 distinct *bla*_ADC_ alleles and only a single *bla*_OXA_ allele, while ST187 and ST23 are associated with eight and seven distinct *bla*_ADC_ alleles, respectively, but only two distinct *bla*_OXA_ alleles each (Table S24).

The majority of STs appear closely associated with both *bla*_ADC_ and *bla*_OXA_ alleles: of 809 STs with isolates encoding assigned *bla*_OXA_ alleles, only 15 were associated with three or more distinct alleles, 62 were associated with just two distinct alleles, and 732 (90.5%) were associated with only a single distinct allele. Of 589 sequence types associated with assigned *bla*_ADC_ alleles, 35 are associated with three or more distinct alleles, 40 with two distinct alleles, and 514 (87.3%) are associated with only a single distinct allele (Table S24).

Alleles appear less closely associated with specific sequence types: *bla*_ADC-156_ is associated with 38 STs, *bla*_ADC-26_ with 37 STs, *bla*_ADC-30_ with 33 STs, *bla*_OXA-65_ with 55 STs, *bla*_OXA-66_ with 52 STs, and *bla*_OXA-69_ with 48 STs. More broadly, 92 *bla*_ADC_ alleles are associated with three or more STS, 29 with two STs, and 82 with only a single ST, while 85 *bla*_OXA_ alleles are associated with three or more STs each, 42 with two STs, and 142 with only a single ST (Table S25).

Among PDS clusters, the most “diverse” cluster is associated with 10 distinct *bla*_ADC_ alleles and 10 distinct *bla*_OXA_ alleles, only 26 clusters are associated with three or more *bla*_ADC_ alleles, five clusters with three or more *bla*_OXA_ allele, and 1,342 clusters (96.1%) are associated with only a single *bla*_OXA_ allele. Interestingly, PDS clusters are not always more closely associated with smaller numbers of alleles compared to sequence types: 1,103 clusters (79.2%) are associated with only a single *bla*_ADC_ allele, roughly 8% lower than by MLST (Table S26). The most frequent alleles tend to be associated with far more clusters than sequence types—*bla*_ADC-30_ is found in 237 clusters, *bla*_ADC-73_ in 122 clusters, *bla*_OXA-66_ in 425 clusters, and *bla*_OXA-69_ in 126 clusters. Conversely, less common alleles do not follow this trend—75 *bla*_ADC_ and 45 *bla*_OXA_ alleles are associated with three or more clusters, 26 *bla*_ADC_ and 17 *bla*_OXA_ alleles are associated with two clusters, and 47 *bla*_ADC_ and 54 *bla*_OXA_ are associated with only a single cluster (Table S27).

#### MLST and PDS granularity

Perhaps not surprisingly, given the difference in resolution between MLST and the wgMLST scheme used to determine PDS clusters, the clusters provide more granularity, resulting in a more diverse and likely more representative, deduplicated data set. Single STs often correspond to many clusters while the converse is generally not true. The most frequent ST, ST2 (63.7% of isolates), corresponds to 482 distinct PDS clusters and additional 22 STs each correspond to 10 or more clusters each. In contrast, the most frequent PDS cluster (10.6% of isolates) corresponds to just seven STs and, in total, only 60 of 1,386 clusters (4.3%) correspond to more than one ST (Table S28).

### BioProject

BioProjects link isolates collected as part of the same studies and often correspond to selection criteria based on phenotype, infection, geography, time, or other characteristics that lead to the collection of many related isolates, potentially influencing the results of our analysis. The largest BioProject varies greatly by region, ranging from 1,882 (North America) to 9 isolates (Oceania). Interestingly, the median number of isolates belonging to a BioProject is between one and three across all regions, suggesting isolates were originally collected for a wide variety of studies in each region (Table S29).

Examining the presence of individual alleles by BioProject, the overall ranks of *A. baumannii* alleles remain largely unchanged, with the two most common alleles *bla*_ADC-30_ (260 BioProjects) and *bla*_ADC-73_ (241 BioProjects) switching places compared to the full data set. The most common intrinsic *bla*_OXA_ allele remains unchanged: *bla*_OXA-66_ (418 BioProjects) while the second most common (*bla*_OXA-95_) drops to 21st most common, suggesting it is highly overrepresented in a small number of BioProjects. Among the 10 most common alleles, the greatest changes are *bla*_ADC-222_ decreasing from third to 24th most common, *bla*_ADC-56_ decreasing from ninth to 17th most common, *bla*_ADC-80_ increasing from 29th to 10th most common, *bla*_OXA-95_ decreasing from second to 21st most common (Table S30).

### Amino acid conservation

Combining the sequences of assigned *bla* alleles present in the Reference Gene Catalog with those of unassigned alleles present in the data set provides a substantial snapshot of the currently known diversity and enables an examination of amino acid conservation of β-lactamase alleles in *A. baumannii*.

#### Conservation among *bla*_ADC_ Variants

**Fig 5:**
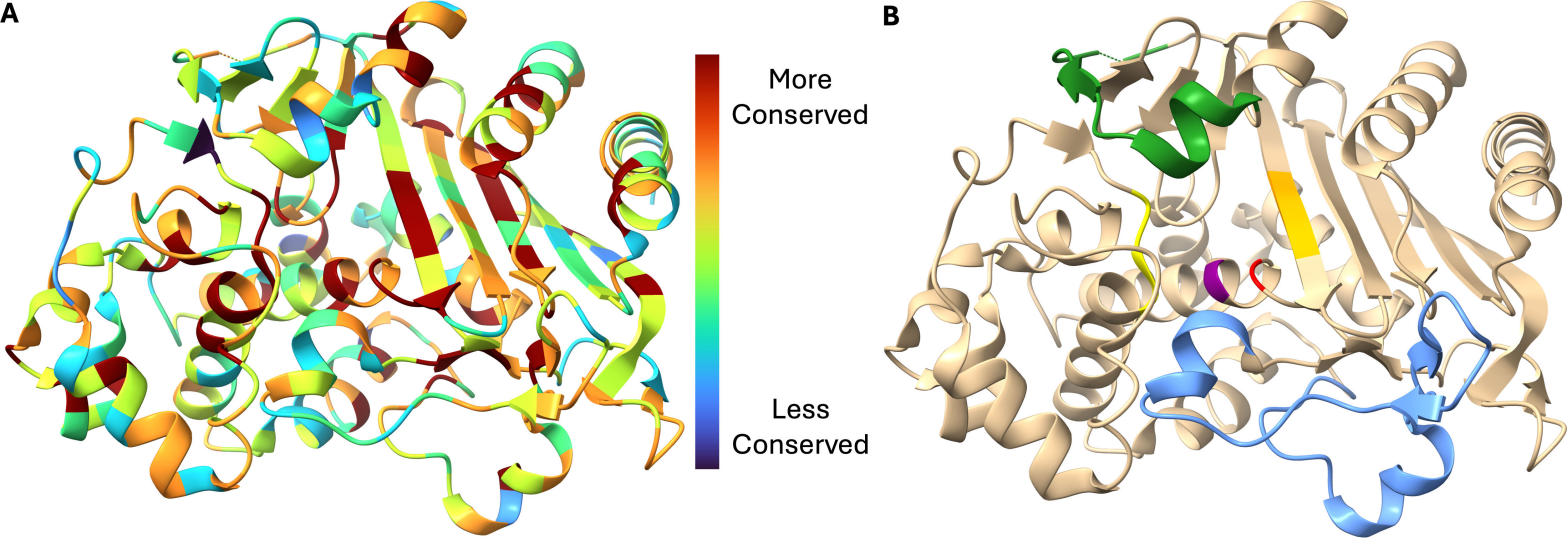
Residue-by-residue conservation and important regions and motifs of *bla*_ADC_ alleles in *A. baumannii*. (**A**) Color-coded conservation of amino acid residues. (**B**) Important regions and motifs of class C β-lactamases, including the catalytic serine (S64; red), general base lysine (K67; purple), general base tyrosine (Y150, as part of the YSN motif; yellow), the Ω-loop (blue), the R2-loop (forest green), and the KTG motif (golden orange). Visualized on the crystal structure of ADC-30 using PDB ID 8FQV (30).

Determining amino acid conservation among the distinct *bla*_ADC_ alleles and using the results to color-code crystal structures provides a residue-by-residue overview of conservation in ADC (Fig. 5). As a companion, Fig. 5B highlights characteristic regions and important residues of class C β-lactamases (using SANC [structural alignment-based numbering of class C β-lactamases] numbering), including the catalytic serine (S64), the general base lysine (K67), the YSN motif (containing the general base Y150), the Ω-loop (approximately residues 188–221), the R2-loop (approximately residues 289–307), and the KTG motif (containing K315) (27–29).

Essential class C β-lactamase catalytic residues and characteristic motifs (S64-V65-S66-K67, Y150-S151-N152, and K315-T316-G317) (27, 28) are highly conserved, along with important residues in the C_3_/C_4_ carboxylate recognition region and core portions of many α-helices. Lower conservation residues are scattered throughout, notably including portions of both the Ω-loop and R2-loop—regions involved in substrate specificity and commonly associated with substrate expansion to hydrolyze newer, larger, or otherwise non-native β-lactams and increased resistance β-lactamase inhibitors (27, 29). Examining ADC protein sequences, nearly all alleles are within 90% identity to all other alleles.

#### Conservation among *bla*_OXA_ variants

**Fig 6:**
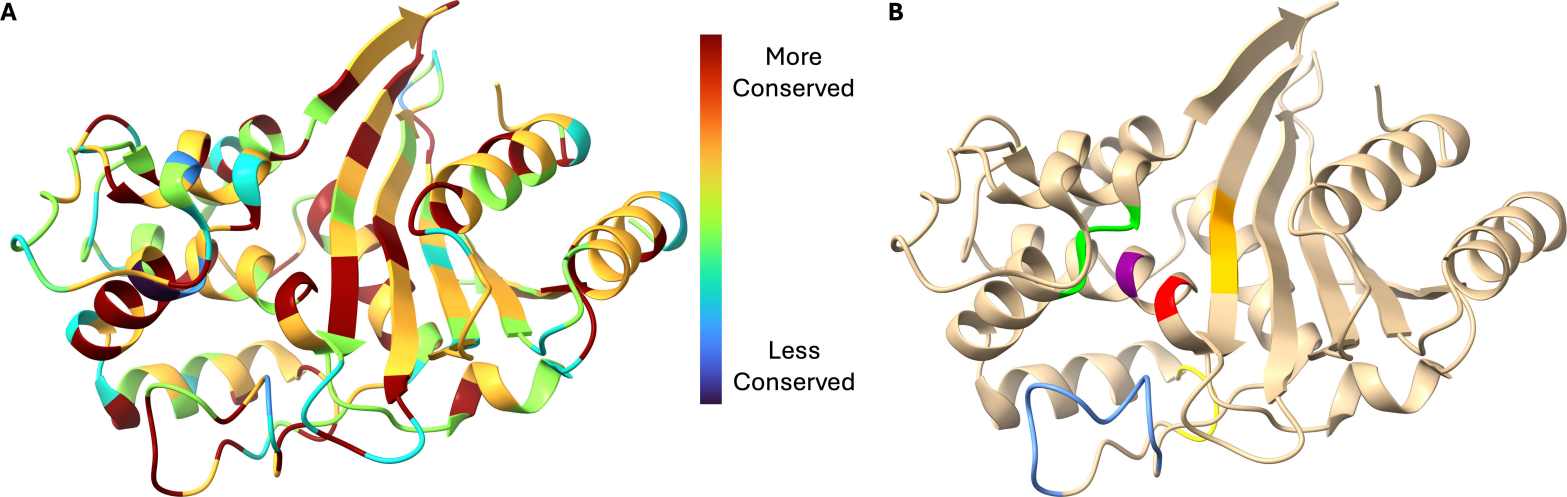
Residue-by-residue conservation of bla_OXA-51_ family member alleles in *A. baumannii*. (**A**) Color-coded conservation of amino acid residues. (**B**) Important regions and motifs of class D β-lactamases, including the catalytic serine (S70; red), general base lysine (K73; purple), the SXV motif (lime green), the (Y/F)GN motif (yellow), the K(T/S)G motif (golden orange), and the Ω-loop (blue). Visualized on the crystal structure of OXA-66 using PDB ID 6T1H (33).

A similar analysis of *bla*_OXA-51_ family members produces a residue-by-residue overview of amino acid conservation. Figure 6A and B highlights important regions and residues of class D β-lactamases, including the catalytic serine (S70), general base lysine (K73), the SXV motif, the (Y/F)GN motif, the K(T/S)G motif, and the Ω-loop (residues 160–169 by the OXA-66 crystal structure; approximately residues 152–167 by DBL [class D β-lactamase] numbering, some of which are not present in OXA-66) (28, 31, 32).

Essential class D β-lactamase catalytic residues (or biochemical properties of the residues) and characteristic motifs (S70-T71-F72/Y72-K73, S118-X119-V120/I120, Y144-G145-N146, and K216-T217/S217-G218) (28, 34) are generally well conserved. Note that the apparent low conservation of residue K216 arises from the way the algorithm handles even rare substitutions with very different amino acid properties (in this case, OXA-1075 contains a K216E substitution)—only one variant has a substitution at this position, and it is, in general, extremely well conserved.

## DISCUSSION

### Insights into the β-lactamase resistome

Significant β-lactamase diversity remains in *A. baumannii*. Despite being a well-studied organism overall, we encountered more than three-and-a-half times as many distinct unassigned *bla*_ADC_ alleles (728) as distinct, assigned alleles (203), suggesting significant unappreciated diversity exists within the β-lactamase resistome (i.e., alleles present in isolates not being submitted for allele assignment or microbiologically/biochemically characterized). Furthermore, only encountering 203 of 322 distinct, assigned alleles present in the Reference Gene Catalog could suggest that many of these alleles may be extremely rare, have evolved, and greatly decreased in frequency since their original isolates were found, or are concentrated in countries where medical/scientific resources are limited and access to genome sequencing is difficult. Considering that allele assignments must be requested by those submitting sequences to NCBI (35), the large number of unassigned alleles are likely unstudied and represent a large reservoir from which future alleles of concern may arise. We believe better understanding of these alleles and fully characterizing those with the most resistant phenotypes should remain a priority of β-lactamase research. Despite the existence of over 1,200 named OXA alleles and 300 named ADC alleles at the time of analysis, there is a broad array of uncharacterized OXA and ADC diversity, even in a well-studied organism such as *A. baumannii*.

A concerning *bla*_ADC_ allele is common in North America. The fourth most common *bla*_ADC_ allele both overall and in North America, *bla*_ADC-33_, has been shown to provide high-level resistance to ceftazidime, cefepime, cefiderocol, and aztreonam (30, 36). Beyond North America, *bla*_ADC-33_ is eighth most common allele in Europe & Central Asia, 13th most common in Middle East & North Africa, 33rd most common in East & Southeast Asia, and was not present in Oceania, South America, South Asia, or Sub-Saharan Africa. Adding an additional element of concern, 662 of 979 isolates (67.6%) encoding a *bla*_ADC-33_ allele also encode a *bla*_OXA-23_ family allele, together likely providing resistance to a large swath of β-lactam antibiotics.

The South American *A. baumannii* β-lactamase resistome continues to differ greatly from other regions. The 1st, 7th, and 10th most frequent *bla*_ADC_ alleles in South America (*bla*_ADC-182_, *bla*_ADC-183_, and *bla*_ADC-235_, respectively) are not found in any other region while two of the four most common *bla*_ADC_ alleles overall, *bla*_ADC-222_ and *bla*_ADC-33_, were not found in any South American isolates. The third and fifth most frequent acquired *bla*_OXA_ alleles in South America (*bla*_OXA-253_ [*bla*_OXA-143_ family] and *bla*_OXA-366_ [*bla*_OXA-23_ family]) were not found in any other region. Several of the most common STs in South America are far less common in other regions (ST79 is the most common in South America but 10th to 32nd in five other regions and not found in two regions, while ST15 is fourth most common in South America but 13th to 19th in four other regions and not found in three regions), suggesting the population structure (not just allele frequencies) is different in the region. Interestingly, alleles found exclusively in South America are distributed both among STs that are exclusive to or highly overrepresented in the region and STs that are relatively common in other regions, meaning differences in ST frequency alone cannot explain the differences in allele distribution. Collectively, these findings align with previous reports that *A. baumannii* in South America can differ substantially from other parts of the world (20, 26, 37, 38), both in terms of β-lactamase alleles and sequence types, helping to verify that our methodology can capture known differences in the population. One may speculate that the uniqueness of the β-lactamase resistome thus may originate from a combination of both differences in the population structure of the strains present in the region and some form of horizontal gene transfer, but we have not explored the root cause of these differences.

Carbapenemase prevalence is high and increasing. As broad spectrum, “last resort” antibiotics for complicated and multidrug-resistant infections (39), carbapenems are an important tool in the clinical armamentarium. Unfortunately, the defining characteristic of CRAb infections is carbapenem resistance, and understanding the diversity and prevalence of these enzymes is crucial to overcoming these infections.

Perhaps, not surprisingly, the frequency of acquired carbapenemases in *A. baumannii* is quite high across all regions (ranging from 67.0% in Europe and Central Asia to 92.2% in Middle East & North Africa), driven primarily by the widespread presence of acquired *bla*_OXA-23_ family alleles. Disturbingly, the presence of carbapenemases has greatly increased over the past 2 decades. Compared to isolates collected from 2000 to 2014, acquired carbapenemase prevalence is roughly 45% higher for isolates collected in 2024. Similar observations around increasing carbapenem prevalence over time have been reported previously (40), but further work is needed to understand the cause of this increase and determine how to best address it.

Understanding the most frequently encountered combinations of β-lactamase alleles provides important insight for research and development. Given that some combinations of enzymes can overcome otherwise effective drug combinations (41), partnering the correct β-lactam and β-lactamase inhibitor is essential, and a detailed overview of allele combinations can help inform that decision. This becomes particularly important when combinations of extended-spectrum AmpC alleles such as *bla*_ADC-33_ occur in combination with serine carbapenemase alleles (such as *bla*_OXA-23_), potentially rendering multiple classes of β-lactamase antibiotics ineffective.

The distribution of β-lactamase alleles is heavily skewed, and most are uncommon. In examining data from 28,330 isolates, we only encountered 203 of 322 assigned *bla*_ADC_ alleles (63.0%). Of these, the majority are extremely infrequent, with 103 *bla*_ADC_ alleles (50.7%) found in 10 or fewer isolates each. Conversely, *bla*_ADC-73_ and *bla*_ADC-30_ alone accounted for 53.3% of all *bla*_ADC_ genes present. Similarly, *bla*_OXA-66_ accounted for 61.2% of all assigned, intrinsic *bla*_OXA_ alleles encountered while 203 of 269 alleles (75.5%) were encountered 10 or fewer times each.

### Breadth versus depth of allelic diversity

For a variety of reasons (likely including the goals of the studies for which isolates were originally collected, outbreaks, and the presence of “successful clones”), some closely related isolates may be overrepresented in the final data set. While this may reflect overall population trends, it runs the risk of masking important, emerging alleles and deemphasizes considerations around similar alleles arising or being disseminated into multiple lineages. One way to think of it is the “depth” (allele frequency) overwhelming the “breadth” (distinct alleles) of diversity, potentially causing important alleles to be missed. By examining the presence of individual alleles in the context of closely related isolates (e.g., MLST and PDS clusters) or those collected for common purpose (e.g., BioProject accessions), we can better understand the breadth of allelic diversity without the distraction of depth.

Deduplication by the presence of individual alleles in MLST sequence types and PDS clusters and unclustered isolates generally led to minor changes for most alleles, suggesting the overall data set is relatively representative of what is present and encountered. Changes for specific alleles, however, can be quite large (e.g., *bla*_ADC-222_ decreasing from third most common to 20th and *bla*_ADC-154_ increasing from 34rd most common to fourth by MLST presence), suggesting the concern of “losing” low frequency, but widespread alleles is valid and needs to be taken into account when performing this and similar types of analysis on large resistome data sets. Interestingly, substantially more and larger changes occurred among *bla*_ADC_ alleles than intrinsic *bla*_OXA_ alleles, suggesting the latter are more evenly distributed across sequence types and clusters. Deduplication by BioProject, in contrast, generally led to smaller changes and revealed only one “lost” allele (*bla*_ADC-154_ increasing from 33rd most common to 10th) and one especially significant decrease (*bla*_ADC-222_ decreasing from third most common to 14th). A substantial majority of sequence types correspond to just one or two distinct, intrinsic *bla*_ADC_ and *bla*_OXA_ alleles, in line with the idea that members of a sequence type are relatively closely related.

PDS clusters provide a far more granular picture of isolates than MLST sequence types. The most frequent sequence type in the data set corresponds to 482 distinct PDS clusters, while the most frequent cluster corresponds to just seven STs. Although not entirely surprising, given the wgMLST origin of PDS clusters (e.g., because of the much higher “resolution” of data being used to define them), the extent of this difference is interesting.

Considering that sequence types can encompass a diverse set of isolates with widely different resistomes and provide only low-resolution data regarding the relatedness of isolates, we support the use of wgMLST based methods going forward. The higher granularity of PDS clusters and other wgMLST outputs is likely to provide a more meaningful understanding of the bacterial populations being examined and will help make useful distinctions when dealing with isolates falling into especially large and/or widespread sequence types, such as the aforementioned ST2 of *A. baumannii*. Conveniently, PDS clusters are included in the MicroBIGG-E database while STs need to be determined by the researcher using sequencing data, making the more granular option more readily available for those working with Pathogen Detection data.

### Comparisons to surveillance studies

The Tigecycline Evaluation and Surveillance Trial (TEST) collected 3,295 meropenem-resistant *A. baumannii* isolates from 47 countries from 2012 to 2016, of which 326 representative strains were subjected to WGS, including 313 with confirmed phenotypes used in the analysis (26). No isolates belonging to the corresponding BioProject (PRJEB27899) were included in our data set as they are not currently present in MicroBIGG-E. The following comparisons are to isolates included in our analysis collected from 2012 to 2016.

Müller and colleagues found ST2 (176 isolates, 56.2%) and ST79 (25 isolates, 8.0%) to be the most common (26), while we found ST2 (911 isolates, 51.5%), ST3 (93 isolates, 5.3%), ST25 (66 isolates, 3.7%), and ST1 (63 isolates, 3.6%) to be the most common. They found *bla*_OXA-23_ family members in 234 isolates (74.8%) and *bla*_OXA-40_ (synonymous with *bla*_OXA-24_) family members in 56 isolates (17.9%) (26), while we found them in 1,150 isolates (65.0%) and 105 isolates (5.9%), respectively. They found *bla*_NDM-1_ in six isolates (1.9%) and *bla*_IMP-26_ in one isolate (0.3%) (26), while we found the former in 47 isolates (2.7%) and did not encounter the latter in isolates collected during this period. Interestingly, we found 84 novel *bla*_OXA-51_ family members (roughly 4.7 per 100 isolates), while they found only two novel *bla*_OXA-51_ family members (roughly 0.6 per 100 isolates) (26).

The Antibiotic Resistance Leadership Group (ARLG) Study Network of *Acinetobacter* as a Carbapenem-Resistant Pathogen (SNAP) examined CRAb isolates collected from 842 patients from around the world between 2017 and 2019 (42). As the SNAP sequences are available in NCBI databases, 153 isolates belonging to the SNAP BioProject (PRJNA906166) are included in our study, collectively representing 1 of 1,093 isolates from 2017, 21 of 1,890 isolates from 2018, and 131 of 3,029 isolates from 2019. These isolates have been excluded from our results for the following comparisons.

The SNAP study found the most common sequence type overall to be clonal group 2 (CG2; corresponding to ST2 and closely related STs; 71.0%). In the USA, CG2 was found in 58.5% of isolates, followed by CG499 (20.9%) and CG406 (8.4%) (42). In our study, overall results are similar with ST2 (3,872 isolates, 66.1%) being the most common, but our results for the USA begin to diverge after the most common (ST2, 573 isolates, 60.8%), with the second most common (ST499, 172 isolates, 14.7%), and third most common (ST406, 43 isolates, 3.7%) representing substantially lower proportions of the total, likely the result of our consideration of only the central ST of each CG as opposed to all members of the CG. Both studies find South or South-Central America to have a very different population structure compared to the rest of the world: the SNAP study found CG1 and CG25 (both 21 isolates or 28.4%) followed by CG15 and CG79 (both 11 isolates or 14.9%) to be the most common in South-Central America (42), while we found ST1 (28 isolates, 29.8%), ST79 (19 isolates, 20.2%), ST25 (18 isolates, 19.1%), and ST15 (nine isolates, 9.6%) to be the most common in South America. They found an acquired carbapenemase gene (defined as a non-*bla*_OXA-51_ family carbapenemase) in 769 (91.3%) of 842 isolates (42), whereas we found 1 (or more) in 4,919 (84.0%) of 5,859 isolates. Among acquired carbapenemase containing isolates, they found *bla*_OXA-23_ in 88.4% of isolates (42) compared to 85.0% of isolates in our study and *bla*_OXA-24_ in 9.8% of isolates (42) compared to 3.7% of isolates in our study (although we found *bla*_OXA-24_ family members in 10.5% of isolates). The SNAP study also found *bla*_NDM-1_, *bla*_OXA-58_, *bla*_OXA-237_, and a *bla*_OXA-134_ family member (42); we found the same genes, as well as *bla*_GES-14_, *bla*_KPC-2_, *bla*_NDM-2_, *bla*_NDM-4_, *bla*_NDM-5_, *bla*_VIM-5_, and (additional) carbapenem-hydrolyzing members of the *bla*_OXA-23_, *bla*_OXA-24_, *bla*_OXA-58_, *bla*_OXA-134_, and *bla*_OXA-143_ families. Their focus on carbapenem-resistant isolates compared to our more widely encompassing approach easily explains the differences in carbapenemase frequency, and their use of CGs as opposed to STs explains our lower ST proportions. Given the limitations of this comparison, general agreement with the trends presented suggests our methodology is successfully capturing allelic diversity in *A. baumannii*.

### Limitations

Focusing our analysis on a database driven approach, we did not directly analyze genomic (except to determine sequence types) or protein data, meaning (i) allele calls and species determinations are analyzed as presented in the database and have not been checked for alternative allele designations or potential speciation errors; (ii) decisions to exclude incomplete sequences and “lower quality” allele calls were based solely on metadata present in the databases used, potentially including a small portion of alleles with sequencing errors; and (iii) protein expression (including the presence of IS*Aba*1 elements needed for the overexpression and thus carbapenemase activity of certain *bla*_ADC_ and *bla*_OXA_ alleles) and resistance phenotype of the isolates encoding these alleles were not considered.

Additionally, while our analysis provides a snapshot of allelic diversity across time and place, it is not a surveillance study and, compared to the rigid selection criteria of a surveillance study, selection bias may be present. The isolates selected for sequencing were chosen to meet the needs and interests of the original researchers, most of which were likely not collected as consecutive isolates or in another unbiased manner. This effect is inherent to the methodology and cannot be readily quantified. Similarly, approximately 95% of isolates, for which source information is available, are clinical samples, meaning they provide a glimpse at what is present in the population but not necessarily what is present in the environment. Indeed, *A. baumannii* is a common environmental and zoonotic organism as well, which may serve as a source of novel alleles (43) that are likely to be underrepresented in the data set.

### Conclusions

By examining the frequency and distribution of β-lactamase alleles in *A. baumannii*, we have determined important alleles and combinations that warrant coverage by future antibiotics and inhibitors and that clinicians should be aware of. We are using these results to prioritize our characterization of enzyme variants and to select targets for ongoing inhibitor development efforts. While the broadly diverse nature of this data set is not ideal for examining evolution or selective pressure, we hypothesize that the presence of extremely successful enzymes may suggest an evolutionary advantage worthy of further study.

As these results demonstrate, substantial, unstudied, and unrecognized allelic diversity remains among β-lactamases in *A. baumannii*. Given the apparent ease with which β-lactamases can evolve expanded-spectrum phenotypes, it is reasonable to speculate that these and other variants could easily serve as a source of antibiotic resistance alleles for future outbreaks when the selective pressure is right. Continued microbiological, biochemical, and evolutionary biology studies are required to understand the ramifications of this diversity. We believe understanding the breadth and depth of this diversity will guide future research and development efforts by focusing on the most frequent and widespread resistance alleles of today and to begin to predict the potential threats of tomorrow.

## MATERIALS AND METHODS

### Data sources and processing

Data were obtained from the NCBI, primarily from databases developed by the Pathogen Detection Program (https://www.ncbi.nlm.nih.gov/pathogens).

Reference Gene Catalog release “2024-07-22.1” (44) was downloaded from the NCBI FTP server (https://ftp.ncbi.nlm.nih.gov/pathogen/Antimicrobial_resistance/Data/2024-07-22.1/ReferenceGeneCatalog.txt) and used to supplement allele and OXA family names as needed.

Data from the Microbial Browser for Identification of Genetic and Genomic Elements (MicroBIGG-E [45]) was obtained on 9 September 2024 using the Google Cloud Platform interface (https://www.ncbi.nlm.nih.gov/pathogens/docs/microbigge_gcp/) with the query “SELECT * FROM ‘ncbi-pathogen-detect.pdbrowser.microbigge’ WHERE (‘taxgroup_name’ like ‘*Acinetobacter baumannii*’) AND (‘element_symbol’ like ‘%*bla*%’).”

We refer to alleles as “assigned” or “unassigned” based on the presence of a formal allele designation. “Assigned” alleles have designation assigned by NCBI (or Institut Pasteur for *bla*_LEN_, *bla*_OKP-A_, *bla*_OKP-B_, and *bla*_OXY_) and are included in the Reference Gene Catalog while “unassigned” alleles lack a formal designation and are not included the Reference Gene Catalog. Assigned alleles include a gene name and number (e.g., *bla*_ADC-7_), while unassigned alleles include only a gene name (e.g., *bla*_ADC_).

Identical Protein Group (IPG) accessions were determined for all alleles appearing in MicroBIGG-E and used to group identical unassigned alleles and to update missing allele assignments for alleles added to the Reference Gene Catalog after isolates were processed. IPG identifiers were determined by querying protein accession numbers using the NCBI Entrez Eutils interface on 9 September 2024, via the REntrez R package.

To help account for potential sequencing errors and differences in sequence quality, we removed “lower quality” allele calls (those with less than 100% coverage, crossing contig boundaries, containing mistranslations or internal stop codons, or unassigned alleles with less than 90% identity to a relevant assigned allele) from the data set. We also removed alleles identified only by hidden Markov models in AMRFinderPlus (46, 47), as they typically do not allow for a complete analysis. As a final quality control step, we removed isolates that did not contain both a *bla*_ADC_ allele and a *bla*_OXA-51_ family allele as these are these intrinsic enzymes are characteristic of *A. baumannii*.

The compiled, processed, and cleaned data set has been uploaded to Zenodo as Table S1, available from https://doi.org/10.5281/zenodo.13841499.

### Data analysis

Data analysis was conducted in RStudio version 2024.04.2 Build 764 using R version 4.4.1.

MLST determinations were made using FastMLST version 0.0.16 (48) with sequences downloaded from NCBI (49). Allele and sequence type definitions were downloaded from PubMLST (50) on 10 September 2024. We utilized the Pasteur scheme (51), which has been described as “more appropriate for population biology and epidemiological studies of *A. baumannii*” (52).

Original code has been prepared as the R package “pdallele.” A snapshot of the version used herein is available at https://doi.org/10.5281/zenodo.13887199 and the most recent version is available at https://github.com/armack/pdallele/.

### Data notes

NCBI databases utilize International Nucleotide Sequence Database Collaboration (INSDC) “country” controlled vocabulary (53), which results in the inclusions of oceans, seas, and territories and regions as “countries,” despite not typically being considered so. We have processed these country names using the “countrycode” R package, resulting in the dropping of location data for ocean/sea locations and some shared or disputed islands from the analysis. Regions (Table S31) are defined based on groupings of United Nations geoscheme subregions (54). We utilized these regional groupings primarily to provide for the separation of North and South America (which most existing United Nations and World Health Organization regions do not), as substantial differences in β-lactamase distribution between these areas have previously been observed (3, 37).

As Pathogen Detection results do not explicitly report chromosomal and non-chromosomal alleles for most isolates (i.e., those not assembled at the “complete genome” level), and most of the assemblies do not have chromosomal vs plasmid contigs annotated as such, we assume that *bla*_ADC_ and *bla*_OXA-51_ family alleles are chromosomal while all other β-lactamase alleles are acquired.

All gene and allele names, *bla*_OXA_ families, and carbapenemase designations as well as species determinations are used and discussed as provided in the NCBI source data. We have not examined the underlying genome or nucleotide data, except to determine sequence types using the PubMLST database.

### Alignments and amino acid conservation

Sequences of assigned *bla*_ADC_ and *bla*_OXA-51_ family alleles appearing in the Reference Gene Catalog and corresponding unassigned alleles appearing in MicroBIGG-E were downloaded as FASTA files using Identical Protein Group identifiers. Multiple sequence alignments were prepared in UniPro UGENE v50.0 (55) using MUSCLE3 (56) with default settings. Amino acid conservation scores were determined in UCSF ChimeraX version 1.8 (57) using independent counts frequency estimation and entropy-based conservation measure with the AL2CO (58) algorithm. Conservation scores were used to color-code the 8FQV crystal structure of ADC-30 (30) and the 6T1H crystal structure of OXA-66 (33) from the RCSB Protein Data Bank (59).

## SUPPLEMENTAL MATERIAL

Figure S1 aac.00784-24-s0001.pdf Regions used in the analysis and isolate counts for each region. Regions could not be assigned for 1,515 of 28,330 isolates.

Supplemental text aac.00784-24-s0002.docx Analysis of allele presence deduplicated data.

Supplemental tables aac.00784-24-s0003.xlsx Tables S2 to S31.

ASM does not own the copyrights to Supplemental Material that may be linked to, or accessed through, an article. The authors have granted ASM a non-exclusive, world-wide license to publish the Supplemental Material files. Please contact the corresponding author directly for reuse.
