# Supplementary material for: β-Lactamase diversity in Acinetobacter baumannii
Source: Antimicrob Agents Chemother. 2025 Feb 10;69(3):e00784-24. doi: 10.1128/aac.00784-24 (PMC11881555; doi:10.1128/aac.00784-24)
Supplement: Figure S1 — Regions used in the analysis and isolate counts for each region. Regions could not be assigned for 1,515 of 28,330 isolates. [file aac.00784-24-s0001.pdf]

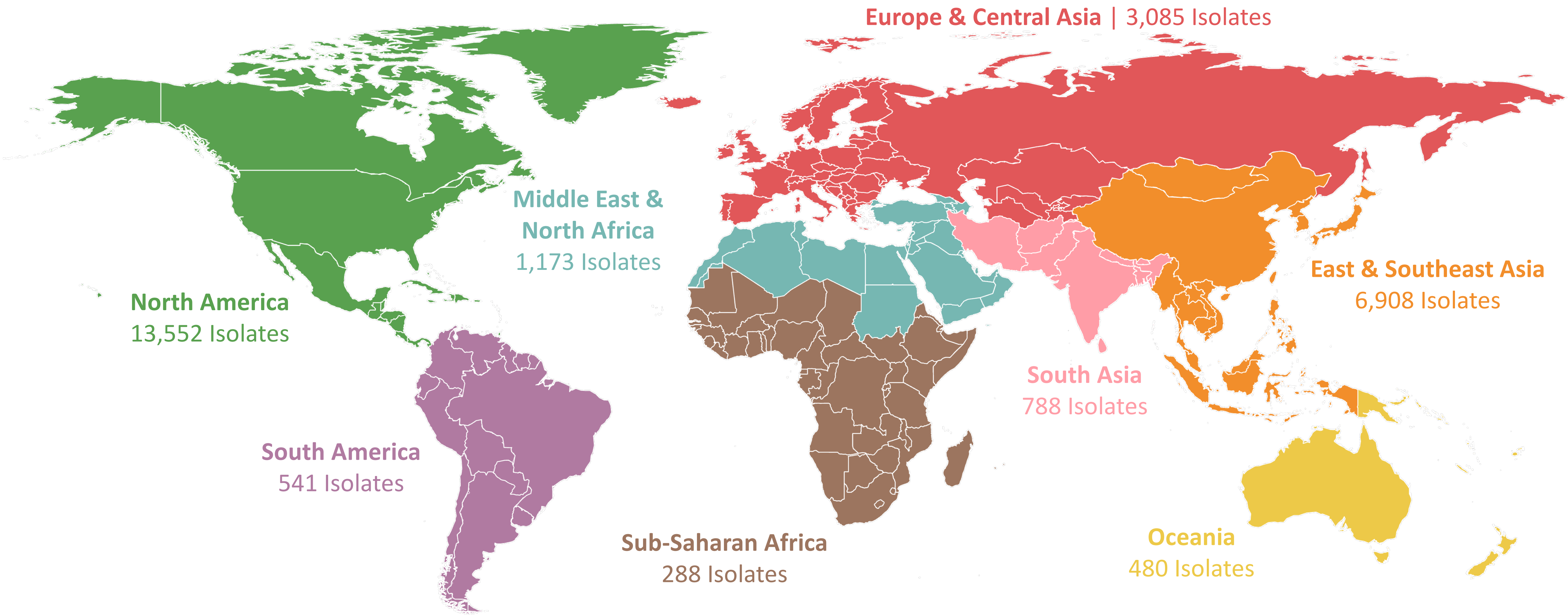

Figure S1. Regions used in the analysis and isolate counts for each region. Regions could not be assigned for 1,515 of 28,330 isolates.
