# Supplementary material for: β-Lactamase diversity in Acinetobacter baumannii
Source: Antimicrob Agents Chemother. 2025 Feb 10;69(3):e00784-24. doi: 10.1128/aac.00784-24 (PMC11881555; doi:10.1128/aac.00784-24)
Supplement: Supplemental text — Analysis of allele presence deduplicated data. [file aac.00784-24-s0002.docx]

**Supplemental Material for β-Lactamase Diversity in *Acinetobacter baumannii***

Andrew R. Mack, Andrea M. Hujer, Maria F. Mojica, Magdalena A. Taracila, Michael Feldgarden, Daniel H. Haft, William Klimke, Arjun B. Prasad, and Robert A. Bonomo

## Overrepresentation of Closely Related Isolates

Given the nature of our analytical approach utilizing an extensive data set originally collected for many different studies, there exists a distinct possibility that some closely related isolates are overrepresented in the data set, which may skew the results. Herein, we endeavor to reduce this effect by examining the binary presence or absence (as opposed to frequency) of distinct alleles among closely related groups of isolates. This approach helps to mitigate the impact of successful clones and outbreaks while amplifying the signal of widespread but low frequency alleles. For purposes of comparison, the “greatest differences” refer to alleles moving by at least six ranks and appearing among the twelve most common alleles for at least one set of conditions.

Examining the presence of individual *bla*_ADC_ alleles by MLST, the most common alleles are: *bla*_ADC-156_ (37 STs, 6.3%), *bla*_ADC-26_ (36 STs, 6.1%), *and bla*_ADC-30_ (32 STs, 5.4%). An additional 36 alleles occur in 1.0% to 5.0% of STs (**Table S23**). By presence in PDS clusters and unclustered isolates, the most frequent alleles are: *bla*_ADC-30_ (598 clusters, 13.7%), *bla*_ADC-33_ (304 clusters, 7.0%), *bla*_ADC-73_ (299 clusters, 6.8%), and *bla*_ADC-26_ (252 clusters, 5.8%). An additional 19 alleles occur in between 1.0 and 5.0% of clusters (**Table S24**).

Compared to the full data set, the greatest differences in the presence of individual *bla*_ADC_ alleles by sequence type and cluster are: *bla*_ADC-222_ decreasing from third most common to 20^th^ my MLST, *bla*_ADC-79_ decreasing from sixth most common to 12^th^ by PDS, *bla*_ADC-56_ decreasing from ninth most common to 16^th^ by MLST, *bla*_ADC-268_ decreasing from 12^th^ most common to 20^th^ by MLST and 28^th^ by PDS, *bla*_ADC-169_ increasing from 17^th^ most common to 11^th^ by MLST, *bla*_ADC-155_ increasing from 20^th^ most common to 7^th^ by PDS, *bla*_ADC-156_ increasing from 21^st^ most common to first by MLST and 6^th^ by PDS, *bla*_ADC-158_ increasing from 27^th^ most common to 7^th^ by MLST, *bla*_ADC-32_ increasing from 28^th^ most common to 5^th^ by MLST and 11^th^ by PDS, *bla*_ADC-80_ increasing from 29^th^ most common to 11^th^ by MLST, *bla*_ADC-165_ increasing form 32^nd^ most common to 6^th^ by MLST, *bla*_ADC-154_ increasing from 34^th^ most common to 4^th^ by MLST, *bla*_ADC-2_ increasing form 44^th^ most common to 8^th^ by MLST, and *bla*_ADC-249_ increasing from 46^th^ most common to 12^th^ by MLST,

Examining the presence of intrinsic *bla*_OXA_ alleles by MLST, the most common alleles are: *bla*_OXA-65_ (54 STs, 6.7%), *bla*_OXA-66_ (51 STs, 6.3%), and *bla*_OXA-69_ (47 STs, 5.8%). An additional 17 alleles appear in 1.0% to 5.0% of STs (**Table S23**). Examining presence using PDS clusters and unclustered isolates, the most common alleles are: *bla*_OXA-66_ (1,112 clusters, 21.9%), *bla*_OXA-82_ (456 clusters, 9.0%), *bla*_OXA-69_ (437 clusters, 8.6%), *bla*_OXA-64_ (271 clusters, 5.3%), and *bla*_OXA-65_ (263 clusters, 5.2%). An additional 13 alleles appear in 1.0% to 5.0% of clusters (**Table S24**).

Compared to the full data set, *bla*_OXA_ alleles are more consistent than *bla*_ADC_ alleles and the greatest differences in presence of individual intrinsic *bla*_OXA_ alleles by sequence type and cluster are: *bla*_OXA-95_ decreasing from second most common to 14^th^ by MLST, *bla*_OXA-71_ decreasing from eighth most common to 13^th^ by MLST and 17^th^ by PDS, *bla*_OXA-100_ decreasing from 11^th^ most common to 18^th^ by MLST, *bla*_OXA-120_ increasing from 12^th^ most common to sixth by MLST, and *bla*_OXA-90_ increasing from 15^h^ most common to 9^th^ by MLST.
